# High pretransplant FGF23 level is associated with persistent vitamin D insufficiency and poor graft survival in kidney transplant patients

**DOI:** 10.1038/s41598-023-46889-0

**Published:** 2023-11-10

**Authors:** Jung-Hwa Ryu, Hee Jung Jeon, Ro Han, Hee-Yeon Jung, Myung-Gyu Kim, Kyu Ha Huh, Jae Berm Park, Kyung Pyo Kang, Seungyeup Han, Jaeseok Yang

**Affiliations:** 1 Department of Internal Medicine, Ewha Womans University Seoul Hospital, Seoul, Republic of Korea; 2 Department of Internal Medicine, Hallym University College of Medicine, Seoul, Republic of Korea; 3 Department of Internal Medicine, Gachon University Gil Medical Center, Incheon, Republic of Korea; 4 Department of Internal Medicine, Kyungpook National University Hospital, Daegu, Republic of Korea; 5 Department of Internal Medicine, Korea University College of Medicine, Seoul, Republic of Korea; 6 Department of Surgery, Yonsei University College of Medicine, Seoul, Republic of Korea; 7 Department of Surgery, Seoul Samsung Medical Center, Sungkyunkwan University, Seoul, Republic of Korea; 8 Department of Internal Medicine, Jeonbuk National University Hospital, Jeonju, Republic of Korea; 9 Department of Internal Medicine, Dongsan Medical Center, Keimyung University, Daegu, Republic of Korea; 10 Department of Internal Medicine, Yonsei University College of Medicine, 50-1 Yonsei-ro, Seodaemun-gu, Seoul, 03722 Republic of Korea

**Keywords:** Nephrology, Risk factors

## Abstract

Vitamin D_3_ (25[OH]D_3_) insufficiency and fibroblast growth factor 23 (FGF23) elevation are usually attenuated after kidney transplantation (KT). However, elevated FGF23 may be associated with poor graft outcomes and vitamin D insufficiency after KT. This study investigated the effect of pretransplant FGF23 levels on post-KT 25(OH)D_3_ status and graft outcomes. Serum FGF23 levels from 400 participants of the KoreaN Cohort Study for Outcome in Patients With Kidney Transplantation were measured. Annual serum 25(OH)D_3_ levels, all-cause mortality, cardiovascular event, and graft survival were assessed according to baseline FGF23 levels. Serum 25(OH)D_3_ levels were initially increased 1 year after KT (12.6 ± 7.4 *vs.* 22.6 ± 6.4 ng/mL). However, the prevalence of post-KT vitamin D deficiency increased again after post-KT 3 years (79.1% at baseline, 30.8% and 37.8% at 3 and 6 years, respectively). Serum FGF23 level was decreased 3 years post-KT. When participants were categorized into tertiles according to baseline FGF23 level (low, middle, high), 25(OH)D_3_ level in the low FGF23 group was persistently low at a median follow-up of 8.3 years. Furthermore, high baseline FGF23 level was a risk factor for poor graft survival (HR 5.882, 95% C.I.; 1.443–23.976, *P* = 0.013). Elevated FGF23 levels are associated with persistently low post-transplant vitamin D levels and poor graft survival.

## Introduction

Fibroblast growth factor 23 (FGF23) is an osteocyte-driven hormone stimulated by high phosphate levels to normalize the phosphate level and is a central regulator in renal phosphate excretion and vitamin D (25[OH]D_3_) homeostasis^1^. FGF23 enhances renal phosphate excretion by downregulating the expression of a sodium/phosphate cotransporter NaPi-IIa in the renal proximal tubules^2,3^. Furthermore, FGF23 potently decreases circulating 1,25(OH)_2_D_3_ levels by inhibiting renal 1-α-hydroxylase activity^3,4^. Elevated FGF23 levels have been documented at the early stage of chronic kidney disease (CKD)^5^, and increased FGF23 level is associated with CKD progression, risk for initiation of dialysis, higher prevalence of cardiovascular disease (CVD), and mortality in patients with CKD^6–9^. Previous studies have reported that high serum FGF23 levels are associated with CVD events and all-cause mortality in the general population^10–13^.

Kidney transplantation (KT) resumes the normal phosphate handling system; accordingly, serum FGF23 level decreases after KT^14^. However, hypophosphatemia and hypercalcemia frequently occur after KT because of persistent elevations in FGF23 and parathyroid hormone (PTH) levels in the early phase after a successful KT^15–17^. Elevated FGF23 levels are closely associated with risk for graft loss, CVD mortality, and all-cause mortality^18,19^. Although high FGF23 levels have been implicated in chronic inflammation, which is correlated with the risk for vascular complications, its precise pathophysiology remains unclear, especially with regarding how FGF23 triggers CVD risk in the KT population. Furthermore, the impacts of the baseline FGF23 levels on post-KT vitamin D levels remain unclear. We investigated the hypothesis that elevated pre-KT FGF23 levels are associated with low vitamin D (25[OH]D_3_) levels and poor long-term post-KT outcomes.

## Methods

### Study design and participants

The KoreaN cohort study for Outcomes in Patients With Kidney Transplantation (KNOW-KT) was a multicenter, prospective, observational cohort study conducted at nine Korean transplantation centers. The study design, methods, and protocol summary have been detailed elsewhere^20^. Briefly, KNOW-KT enrolled Korean patients over 18 years of age who underwent KT, and corresponding donors between 2012 and 2016. The study was conducted in accordance with the principles of the Declaration of Helsinki and the Declaration of Istanbul, and the Institutional Review Boards at Ewha Womans University College of Medicine/Ewha Womans University Hospital approved the study protocol of participating centers (2022-10-064-001).

**Figure 1:**
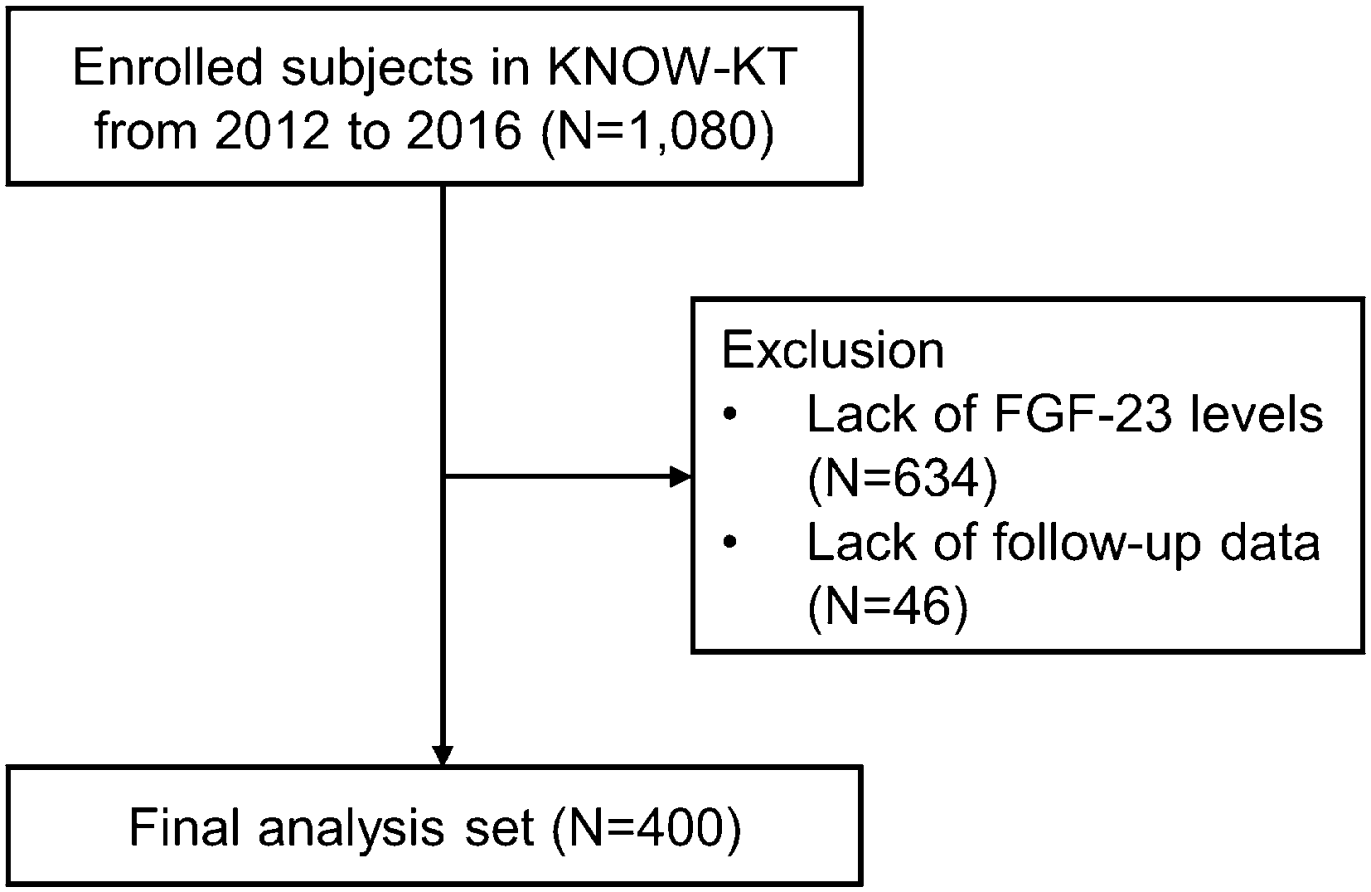
Flowchart of the study population.

Informed consent was obtained from all 1080 subjects and/or their legal guardian. Patients without follow-up (n = 46) and FGF23 (n = 634) data were excluded. The clinical characteristics of the excluded patients were not significantly different from those included in this study (Supplementary Table 1). Ultimately, 400 patients were included in the final analysis (Fig. 1).

### Data collection

Baseline and followed-up data were retrieved using an electronic data management system (PhactaX, Seoul, Republic of Korea). Sociodemographic information, including age, sex, history of smoking and alcohol consumption, cause of end-stage renal disease (ESRD), comorbid diseases, and medications, were collected during the pre-KT screening period. At the time of KT, transplant-related parameters were collected, including the date of transplantation, number of transplant experiences, donor-recipient relationship, and desensitization. Body mass index was calculated as the weight divided by height squared (kg/m^2^). Immunosuppressive medications were recorded at discharge as baseline data, and then at every annual visit.

Laboratory data included serum levels of blood urea nitrogen (BUN), creatinine, calcium, phosphorus, total cholesterol, triglycerides, low-density lipoprotein (LDL) cholesterol, and albumin analyzed by auto analyzer due photometric method. Hemoglobin was measured by Beckman Coulter (DxH900, Beckman Coulter, Brea, CA, USA), glycated hemoglobin was measured by high performance liquid chromatography using HLC-723 G11 analyzer (Tosoh Corporation, Tokyo, Japan), and high-sensitive C-reactive protein (hs-CRP) was measured by Turbidimetric immunoassay using AU-5800 (Beckman Coulter, Brea, CA, USA). These parameters are measured routinely at annual visits. Intact parathyroid hormone (PTH) and serum 25(OH)D_3_ level was measured using ECLIA (electrochemiluminescence immunoassay) method (unicel DXI 800, Beckman Coulter, Brea, CA, USA). These parameters were measured routinely at each annual visit. Immunologic evaluation included human leukocyte antigen (HLA) typing, cross-matching (complement-dependent cytotoxicity-based and flow cytometry-based methods), and panel reactive antibody levels. Intact FGF23^21^ levels were measured using a commercially available enzyme-linked immunosorbent assay kit (Kainos, Tokyo, Japan) at baseline and 3 years post-KT. We used banked samples for FGF23 measurements. The estimated glomerular filtration rate (eGFR) was calculated using the CKD-Epidemiology Collaboration equation^22^.

### Outcomes

The primary outcome of interest was death-censored graft failure, which included restarting dialysis or re-transplantation. The secondary outcomes included all-cause mortality and cardiovascular events.

### Statistical analysis

All continuous variables are expressed as mean ± standard deviation (SD) or median with interquartile ranges (IQR). Categorical variables are expressed as numbers of subjects with percentages. Differences in baseline characteristics were compared among the groups, which were divided according to the baseline FGF23 level, using a one-way analysis of variance. The Kruskal–Wallis test was used for non-normally distributed data. Graft survival according to baseline FGF23 tertiles was analyzed using the Kaplan–Meier method. Multivariate analysis was performed using the Cox proportional-hazard model to assess the association of baseline FGF23 tertiles and death-censored graft failure. To consolidate the results of the primary analysis, a series of hazard models was conducted. The crude model represents unadjusted hazard ratios (HRs); model 1 was adjusted for eGFR at baseline; and model 2 was adjusted for age, sex, CVD, diabetes mellitus, transplant donor type (living or deceased donor), HLA-incompatible transplantation, ABO-incompatible transplantation, acute rejection episode, and factors included in model 1. Model 3 was adjusted for vitamin D supplementation at baseline, dialysis vintage, serum PTH, phosphate, and 25(OH)D_3_ levels, in addition to the factors included in model 2. A linear mixed model equation was used to determine the factors affecting 25(OH)D_3_ levels over 9-year follow up. All statistical tests were two-sided and differences with *P* < 0.05 were statistically significant. Statistical analyses were performed using SPSS, version 27.0 (IBM Corporation, Armonk, NY, USA).

## Results

### Baseline clinical characteristics

Baseline characteristics according to pretransplant FGF23 level tertiles are summarized in Table 1. The mean age of the participants was 45.7 ± 11.3 years, and 64.3% were male. Median baseline serum FGF23 level was 2140.6 (391–9277) pg/ml and categorized into tertile according to median baseline FGF23 levels (low, middle, high) as follows: 178.3 (94.2–393.8) pg/ml; 2140.6 (1379.9–3143.8) pg/ml; and 17,034.4 (9107.7–48,031.4) pg/ml, respectively. According to the FGF23 tertiles, mean age and sex did not exhibit significant intergroup differences. The mean eGFR was 63.7 ± 18.9 ml/min per 1.73 m^2^, which was similar among the groups. The causes of ESRD, comorbidities, and medications were similar among the FGF23 tertiles. Serum 25(OH)D_3_ levels differed according to FGF23 tertiles; patients with lower levels of FGF23 had higher proportions of preemptive, living donor transplantation, shorter dialysis vintage, and higher 25(OH)D_3_ levels compared to those with higher FGF23. Baseline 25(OH)D_3_ level was the lowest in high FGF23 tertile compared to the other groups; 12.9 (8.6–17.6), 12.5 (7.9–17.9), and 9.8 (6.1–13.4) ng/ml in the low, middle, and high tertiles, respectively. Serum calcium level was stepwisely higher according to tertiles; 8.4 ± 0.9, 8.9 ± 0.9, and 9.2 ± 1.5 mg/dl in the low, middle, and high tertiles, respectively. Serum phosphorus level was higher in high FGF23 tertile compared to the other tertile groups; 4.6 ± 1.1, 5.2 ± 1.4, and 5.5 ± 1.5 mg/dl in the low, middle, and high tertile groups, respectively. Although median serum intact PTH level was higher in the high tertile group, the difference was not statistically significant: 208.1 (98.6–334.3), 205.0 (118.5–351.1), and 247.3 (133.8–409.2) pg/ml in the low, middle, and high tertile groups, respectively.

**Table 1:** Baseline clinical characteristics according to baseline FGF23 levels.

	Total (N = 400)	Tertile 1 (n = 133)	Tertile 2 (n = 133)	Tertile 3 (n = 134)	P value
Age (years), mean ± SD	45.7 ± 11.3	45.7 ± 11.3	42.6 ± 11.4	46.2 ± 11.7	0.885
Male gender, n (%)	257 (64.3%)	81 (60.9%)	95 (71.4%)	81 (60.4%)	0.107
BMI (kg/m^2^)	22.8 ± 3.4	22.8 ± 3.5	22.6 ± 3.4	23.0 ± 3.5	0.544
Cause of ESRD, n (%)					0.191
DM	73 (18.3%)	20 (15.0%)	24 (18.0%)	29 (21.6%)	
HTN	64 (16.0%)	12 (9.0%)	25 (18.8%)	27 (20.1%)	
GN	122 (30.5%)	46 (34.6%)	40 (30.1%)	36 (26.9%)	
ADPKD	24 (6.0%)	11 (8.3%)	6 (4.5%)	7 (5.2%)	
Others	31 (7.7%)	10 (7.5%)	8 (6.0%)	13 (9.7%)	
Unknown	86 (21.5%)	34 (25.6%)	30 (22.6%)	22 (16.4%)	
Diabetes mellitus, n (%)	93 (24.2%)	28 (21.9%)	30 (23.3%)	35 (27.6%)	0.543
Hypertension, n (%)	368 (92.0%)	124 (93.2%)	120 (90.2%)	124 (92.5%)	0.140
Cardiovascular disease, n (%)	29 (7.6%)	7 (5.5%)	9 (7.0%)	13 (10.2%)	0.338
Cerebrovascular disease, n (%)	12 (3.1%)	3 (2.3%)	6 (4.7%)	3 (2.4%)	0.474
Type of RRT					0.001
HD	278 (69.5%)	93 (69.9%)	99 (74.4%)	86 (64.2%)	
PD	54 (13.5%)	4 (3.0%)	15 (11.3%)	35 (26.1%)	
Preemptive	64 (16.0%)	35 (26.3%)	16 (12.0%)	13 (9.7%)	
Transplantation	4 (1.0%)	1 (0.8%)	3 (2.3%)	0 (0.0%)	
Dialysis vintage prior to transplant (years), median (IQR)	0.5 (0.1–3.7)	0.2 (0.1–0.6)	0.4 (0.1–1.8)	3.0 (0.4–7.0)	0.001
Donor source, n (%)					0.001
Living	312 (78.0%)	115 (86.5%)	113 (85.0%)	84 (62.7%)	
Deceased	88 (22.0%)	18 (13.5%)	20 (15.0%)	50 (37.3%)	
Desensitization therapy, n (%)	93 (23.3%)	28 (21.1%)	35 (26.3%)	30 (22.4%)	0.572
Immunosuppressant, n (%)					
CNI (Tacrolimus)	391 (97.8%)	131 (98.5%)	129 (97.0%)	131 (97.8%)	0.710
Mycophenolate mofetile	257 (64.3%)	91 (68.4%)	89 (66.9%)	77 (57.5%)	0.128
Mycophenoleic acid	168 (42.1%)	51 (38.3%)	47 (35.3%)	70 (52.6%)	0.010
mTOR inhibitors (sirolimus & everolimus)	64 (16.0%)	23 (17.2%)	29 (21.8%)	12 (9.0%)	0.102
Prednisolone	400 (100.0%)	133 (100.0%)	133 (100.0%)	134 (100.0%)	–
Medication					
RAS blockers	208 (52.0%)	70 (52.5%)	69 (51.9%)	69 (51.5%)	0.692
Statins	137 (34.5%)	46 (34.6%)	47 (35.2%)	44 (32.8%)	0.683
Anti-platelet agents	60 (15.0%)	15 (11.3%)	21 (15.8%)	24 (17.9%)	0.032
Vitamin D supplements	68 (17.0%)	25 (18.8%)	22 (16.5%)	21 (15.4%)	0.055
Laboratory findings					
Serum creatinine (mg/dl), mean ± SD	1.19 ± 0.47	1.21 ± 0.43	1.29 ± 0.60	1.23 ± 0.52	0.378
eGFR (ml/min/1.73 m^2^), mean ± SD	63.7 ± 18.9	63.7 ± 18.5	62.9 ± 18.2	64.5 ± 19.9	0.795
Albumin (g/dL), mean ± SD	4.0 ± 0.5	3.9 ± 0.5	4.0 ± 0.5	4.0 ± 0.5	0.120
Hemoglobin (g/dL), mean ± SD	10.5 ± 1.6	10.2 ± 1.5	10.6 ± 1.6	10.7 ± 1.6	0.098
C-reactive protein (mg/dL), median (IQR)	0.09 (0.04–0.30)	0.08 (0.03–0.3)	0.10 (0.05–0.27)	0.09 (0.03–0.34)	0.405
Total cholesterol (mg/dL), mean ± SD	156.7 ± 39.0	154.9 ± 37.8	156.8 ± 36.1	158.3 ± 43.1	0.776
Triglyceride (mg/dL), mean ± SD	125.2 ± 87.0	123.2 ± 88.2	127.5 ± 83.8	124.8 ± 89.5	0.921
LDL cholesterol (mg/dL), mean ± SD	83.8 ± 29.6	82.7 ± 27.6	83.4 ± 28.3	85.2 ± 32.8	0.791
Calcium (mg/dL), mean ± SD	8.8 ± 0.9	8.4 ± 0.9	8.9 ± 0.9	9.2 ± 1.5	0.03
Phosphorus (mg/dL), mean ± SD	5.2 ± 1.4	4.6 ± 1.1	5.2 ± 1.4	5.5 ± 1.5	0.01
PTH (pg/mL), median (IQR)	205.0 (118.5–351.0)	208.1 (98.6–334.3)	205.0 (118.5–351.1)	247.3 (133.8–409.2)	0.087
FGF23 (pg/mL), median (IQR)	2140.6 (391–9277)	178.3 (94.2–393.8)	2140.6 (1379.9–3143.8)	17,034.4 (9107.7–48,031.4)	0.01
25(OH)D_3_ (ng/mL), median (IQR)	11.3 (7.1–17.0)	12.9 (8.6–17.6)	12.5 (7.9–17.9)	9.8 (6.1–13.4)	0.001

*BMI* body mass index, *ESRD* end-stage renal disease, *DM* diabetes mellitus, *HTN* hypertension, *GN* glomerulonephritis, *ADKPD* autosomal dominant polycystic kidney disease, *RRT* renal replacement therapy, *HD* hemodialysis, *PD* peritoneal dialysis, *IQR* interquartile range, *CNI* calcineurin inhibitor, *mTOR* mouse target of rapamycin, *RAS* renin–angiotensin system, *eGFR* estimated glomerular filtration rate, *PTH* parathyroid hormone, *FGF23* fibroblast growth factor 23, *25(OH)D*_*3*_ 25-hydroxy vitamin D_3_.

### Changes in FGF23 levels after kidney transplantation

Serum FGF23 levels decreased after KT (pre-KT, 2140.6 [391–9277] pg/ml vs. 50.0 [23.6–94.6] pg/ml 3 years after KT, *P* = 0.001) (Fig. S1A). FGF23 levels 3 years after KT correlated well with the pre-transplant FGF23 levels (r^2^ = 0.095, *P* = 0.021) (Fig. S1B).

### Longitudinal change in 25(OH)D_3_ levels during follow-up according to FGF23 tertiles

**Figure 2:**
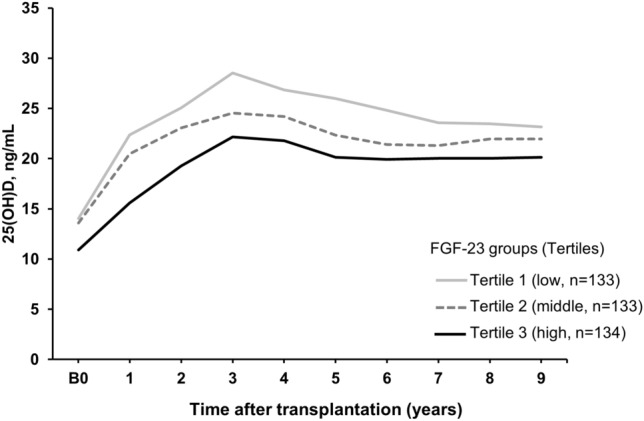
Post-transplant changes in 25(OH)D_3_ levels according to the tertiles of baseline FGF-23 level. The patients were divided into 3 groups according to baseline FGF23 levels: low (n = 133), middle (n = 133), and high FGF23 tertile (n = 134). 25(OH)D_3_ levels were lower in the higher FGF23 tertile group during followup.

25(OH)D_3_ levels increased up to 3 years after KT, decreased, then reached plateau approximately 7 years after KT (Fig. 2). Patients with higher FGF23 levels exhibited low 25(OH)D_3_ levels during the study period (Fig. 2). In the analysis using linear mixed model estimation to identify factors associated with a higher 25(OH)D_3_ levels over the 9-year period, both the high (*P* = 0.015) and middle (*P* = 0.025) FGF23 tertiles were inversely associated with 25(OH)D_3_ levels (Table 2).

**Table 2:** Association of baseline FGF-23 with 25(OH)D_3_ levels over 9 year-follow up.

Parameter	Higher 25(OH)D_3_ level		
	Estimate (95% CI)	SE	*P* value
Time	1.178 (0.871, 1.486)	0.157	< 0.001
Age	0.136 (0.058, 0.214)	0.040	0.005
Gender (male)	1.247 (−0.585, 3.078)	0.585	0.182
Hypertension	1.081 (−2.216, 4.327)	0.426	0.514
Diabetes mellitus	−1.353 (−3.352, 0.647)	1.020	0.185
Cardiovascular Ds	−0.140 (−2.662, 2.942)	1.429	0.792
Cerebrovascular Ds	−1.893 (−5.750, 1.965)	1.968	0.336
Hemodialysis before KT	3.949 (1.706, 6.193)	1.145	0.005
Dialysis vintage	−0.005 (−0.026, 0.017)	0.011	0.663
BMI-B^0^	−0.157 (−0.393, 0.079)	0.121	0.192
eGFR-B_0_	−0.006 (−0.053, 0.040)	0.066	0.797
Albumin	0.761 (−2.024, 4.329)	1.002	0.541
Hemoglobin	0.451 (−0.489, 0.832)	0.312	0.210
Phosphorus	−0.421 (−0.977, 0.135)	0.283	0.138
C-reactive protein	−0.372 (−6.023, 1.132)	0.329	0.562
Deceased donor	−1.251 (−2.982, 0.593)	0.982	0.134
Desensitization before KT	1.665 (−0.253, 3.583)	0.979	0.089
FGF23-B_0_ (vs. low tertile)			
Middle tertile	−2.502 (−4.518, −0.487)	1.028	0.025
High tertile	−2.550 (−4.780, −0.319)	1.138	0.015

*25(OH)D*_*3*_ 25-hydroxy vitamin D_3_, *BMI* body mass index, *eGFR* estimated glomerular filtration rate, *KT* kidney transplantation, *FGF23* fibroblast growth factor 23.

### Association between FGF23 levels and graft failure

**Figure 3:**
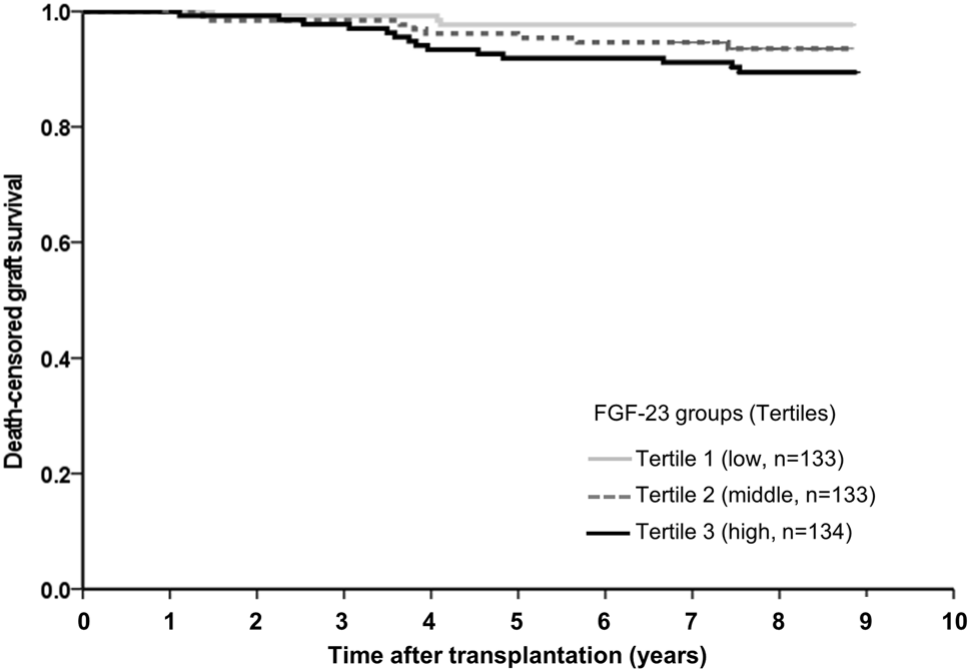
Death-censored graft survival rates according to tertiles of baseline FGF-23 levels. Death-censored graft survival was compared among baseline FGF23 tertile groups. Each group includes 133 patients in low tertile, 133 patients in middle tertile, and 134 participants in high tertile. The allograft survival rate was higher in the group with low FGF23 compared to the lower tertiles (*P* = 0.016 by log rank).

Graft failure developed in 26 (6.5%) patients at a median follow-up of 8.3 (7.9–8.8) years (Table 3). The incidence rates were 1.5%, 6.0%, and 11.9% according to the low, middle, and high FGF23 tertiles (*P* < 0.05) (Table 3), respectively. Graft survival was higher in the high FGF23 tertile than in the low FGF23 tertile (*P* = 0.016) (Fig. 3); in multivariate Cox regression analysis, a higher baseline FGF23 level was an independent risk factor for graft failure; patients in the high and middle FGF23 tertiles were associated with a 5.882-fold (95% C.I., 1.443–23.976, *P* = 0.013) and 2.737 (95% C.I., 0.690–10.855, *P* = 0.152) higher risk for graft failure than those in the low FGF23 tertile in final adjusted model 3 (Table 4).

**Table 3:** Post-transplant clinical characteristics according to baseline FGF23 levels.

Outcomes	Overall (N = 400)	FGF23 categories (pg/ml)			
		Low tertile (n = 133)	Middle tertile (n = 133)	High tertile (n = 134)	*P* value
Death event					
No. of person-years	3315.8	1105.6	1098.2	1112.0	
Incidence of outcomes, n (%)	11 (2.8)	3 (2.3)	1 (0.8)	7 (5.2)	0.073
Incidence rate per 1000 person-year	3.3	2.7	0.9	6.3	
Cardiovascular event					
No. of person-years	3177.8	1061.2	1041.9	1074.7	
Incidence of outcomes, n (%)	24 (6.0)	8 (6.0)	8 (6.0)	8 (6.0)	1.000
Incidence rate per 1000 person-year	7.6	7.5	7.7	7.4	
Cerebrovascular event					
No. of person-years	3288.7	1098.4	1078.3	1112.0	
Incidence of outcomes, n (%)	5 (1.3)	2 (1.5)	3 (2.3)	0 (0.0)	0.242
Incidence rate per 1000 person-year	1.5	1.8	2.7	0	
Acute rejection (ATMR + ABMR)					
No. of person-years	2832.6	931.4	900.9	1000.3	
Incidence of outcomes, n (%)	64 (16.0)	23 (17.3)	27 (20.3)	14 (10.4)	0.232
Incidence rate per 1000 person-year	22.6	24.7	30.0	14.0	
Graft loss					
No. of person-years	3243.9	1096.4	1076.5	1070.9	
Incidence of outcomes, n (%)	26 (6.5)	2 (1.5)	8 (6.0)	16 (11.9)	0.018
Incidence rate per 1000 person-year	8.0	1.8	7.4	15.0	
Fracture					
No. of person-years	3202.1	1059.2	1049.3	1069.2	
Incidence of outcomes, n (%)	18 (4.5)	6 (4.5)	6 (4.5)	6 (4.5)	1.000
Incidence rate per 1000 person-year	5.6	5.6	5.7	5.6	

*FGF23* fibroblast growth factor 23, *ATMR* acute T cell mediated rejection, *ABMR* acute antibody mediated rejection.

**Table 4:** Association of baseline FGF-23 with graft failure.

Model	FGF23 tertiles	Graft failure	*P* value
		HR (95% CI)	
Crude	Low tertile	1	–
	Middle tertile	2.773 (0.778–10.474)	0.131
	High tertile	4.646 (1.335–16.168)	0.016
Adjusted model 1^a^	Low tertile	1	–
	Middle tertile	2.779 (0.737–10.480)	0.131
	High tertile	4.800 (1.379–16.703)	0.014
Adjusted model 2^b^	Low tertile	1	–
	Middle tertile	2.530 (0.658–9.724)	0.097
	High tertile	5.071 (1.377–18.677)	0.015
Adjusted model 3^c^	Low tertile	1	–
	Middle tertile	2.737 (0.690–10.855)	0.152
	High tertile	5.882 (1.443–23.976)	0.013

^a^Adjusted for eGFR at baseline.

^b^Model 1 + adjusted for age, sex, cardiovascular disease, diabetes mellitus, transplantation type (deceased donor transplantation or living donor transplantation), HLA incompatible transplantation, ABO-incompatible transplantation, acute rejection episode.

^c^Model 2 + adjusted for vitamin D supplementation at baseline, dialysis vintage, serum PTH, phosphate, and 25(OH)D_3_.

### Association between FGF23 levels and secondary outcomes

Table 3 shows the post-transplant incidence of various outcomes after KT, including death, CVD, stroke, acute rejection, and fracture. The incidence of death was 2.3%, 0.8%, and 2.2% low, middle, and high FGF23 tertiles, respectively (*P* = 0.556). The incidence of CVD was 6.0% in all FGF23 tertiles (*P* = 1.000). The incidence of all-cause mortality, cardiovascular events, stroke events, acute rejection, and fracture development did not differ according to FGF23 tertiles.

## Discussion

In this study, we investigated the clinical implications of pretransplant FGF23 status on post-transplant long-term outcomes. Higher FGF23 levels were significantly associated with a higher risk of low vitamin D levels and incident graft failure. Higher FGF23 levels at pretransplant was associated with a persistent lower 25(OH)D_3_ level after KT. Furthermore, high FGF23 tertile group exhibited a 5.8 -fold higher risk for graft failure than the low FGF23 tertile group. This association was independent of post-transplant renal function, 25(OH)D_3_ level, phosphate level, HLA- or ABO-incompatible transplantation, and other comorbidities.

Most studies investigating the clinical implications of FGF23 have focused on cardiovascular and all-cause mortality because of its pathophysiologic role in dysregulated mineral bone metabolism associated with cardiovascular damage and CKD progression^23^. The proposed mechanism is that elevated serum levels of FGF23 lead to left ventricular hypertrophy and endothelial dysfunction, worsening arterial stiffness, and accelerated cardiovascular damage in patients with CKD^24–29^. Based on these studies, the harmful effect of FGF23 on the cardiovascular system led to our advanced understanding that FGF23 is not only a signal transducer to handle phosphate handling, but also a feasible biomarker for CVD and all-cause mortality in the CKD population. Even in community-based populations, elevated serum FGF23 level are associated with CVD events or mortality^11^. However, the significance of high FGG23 levels as an independent risk factor for all-cause and CVD mortality was attenuated when adjusted for decreased eGFR or old age^26^.

In patients who have undergone KT, high plasma FGF23 levels are an independent risk factor for cardiovascular-related mortality. This suggests that FGF23 resistance caused by a non-recovery of the mineral bone disease axis causes CVD after KT^19,30^. However, our study could not observe an association between FGF23 levels and CVD or all-cause mortality after KT. A possible explanation is that most of the study population who underwent living donor KT may have had relatively early stage of mineral-bone diseases. Improved renal phosphate excretion and lower blood phosphorus levels after KT may have attenuated an association between FGF23 and advanced CVD risk in KT patients. In parallel, a community-based study involving patients with normal to moderate CKD reported that FGF23 concentrations were correlated with CVD events and mortality only when concurrent lower urinary phosphate excretion was observed^31,32^. Furthermore, the significance of FGF23 as a risk factor for CVD events and mortality has been demonstrated mainly in patients with normal or high blood phosphorus levels^32^. Bienaimé1 et al.^32^ did not find an association between early FGF23 levels after KT and CVD outcomes, which is consistent with our results. This finding suggests that suboptimal tubular responses to FGF23 are more important than serum FGF23 levels in patients with mild renal dysfunction. Unfortunately, urinary phosphate excretion ratio was not measured in this study. Therefore, a larger study with concurrent measurements of serum FGF23 levels and urinary phosphate excretion fractions in the KT population may be required to determine the impact of different tubular functions in response to FGF23.

FGF23 elevation is associated with elevated levels of resistin, an adipocytokine that is primarily expressed in macrophages and leukocytes. Resistin can act as a pro-inflammatory cytokine and be associated with graft loss and death of functioning grafts in a 6-year follow-up study^33^. FGF23 can also activate pro-inflammatory macrophages through the reconstitution of FGFR/α-klotho signaling^34,35^. Higher FGF23 levels during the pre-transplant period may induce a condition prone to inflammation after transplantation independent of renal function. Chronic exposure to high FGF23 levels in patients with CKD may contribute to poor graft outcomes after KT.

Consistent with pro-inflammatory role of FGF23, previous studies have demonstrated that increased pre-transplant FGF23 levels are a significant risk factor for adverse graft outcomes after KT^18,19^. In parallel, this study demonstrated that a high pre-transplant FGF23 level is a robust independent risk factor for poor long-term allograft survival after KT. The effects of FGF23 on graft function differ among studies, depending on the time of FGF23 measurement. The effects of FGF23 on graft survival were not demonstrated in another study that examined post-transplant FGF23 levels at 1 year after transplantation^32^. The present study also measured FGF23 levels at 3 years after KT, as well as pre-transplant FGF23 levels. However, there was no association between post-transplant FGF23 levels and graft survival (data not shown). Why post-transplant FGF23 is a minimal contributor to graft outcome is not clear; however, we speculate that the pro-inflammatory effect of post-transplant FGF23 may not be critical after KT, where the chronic inflammatory milieu under CKD is attenuated. However, the association between higher FGF23 levels and adverse graft outcomes often faded when it was adjusted by eGFR^19,32^. Post-transplant FGF23 concentration is also strongly associated with eGFR at the same time^36^. Whether pre- and post-transplant FGF23 levels exert different effects on graft function should be investigated in future studies.

The present study revealed annual longitudinal changes in 25(OH)D_3_ levels over a 9-year follow-up period according to FGF23 levels. The 25(OH)D_3_ levels tended to be higher in patients taking vitamin D supplements. Although elevated FGF23 concentration at pre-transplant status usually exhibited a trend of prompt decrease along with improved renal function after KT, especially within 3 months^15,37,38^, high pre-transplant FGF23 concentrations can affect long-term graft function. After 3 months, normalization in FGF23, PTH, and the calcium connecting system progresses at a slower rate, although endocrine alterations did not fully recover to homeostasis^39,40^. A possible pathophysiology of this finding was introduced as “tertiary hyperphosphatoninism”, which refers to a condition of autonomous secretion of FGF23 after KT^16^. Evenepoel et al.^16^ showed that high post-transplant FGF23 concentrations were independently associated with high pre-transplant FGF23 levels, which may suppress the recovery of calcitriol after transplantation. We found that 25(OH)D_3_ levels were persistently lower during the 9-year follow-up period in the high FGF23 tertile. The effect of baseline FGF23 levels on long-term 25(OH)D_3_ metabolism has not yet been determined. High FGF23 levels can activate 24-hydroxylase and increase metabolic degradation of 25(OH)D_3_ and 1,25(OH)_2_D_3_^41^. High pre-transplant FGF23 levels could disrupt the physiological functional recovery of calcitriol, PTH, and phosphate-interrelating system. High FGF23 levels induce a 1,25(OH)_2_D_3_ deficiency, which could contribute to immunologic dysregulation in allografts^42–44^. This speculation is supported by the study results, which demonstrated the harmful impact of 25(OH)D_3_ deficiency on graft failure and the beneficial effect of vitamin D supplementation on allograft outcomes^45–47^. Based on this evidence, suboptimal active 25(OH)D_3_ levels may be associated with FGF23 resistance caused by inappropriately prolonged high FGF23 levels after KT. Our results extend the findings of previous investigations addressing FGF23 and graft loss in those with persistent vitamin D deficiency in KT recipients with higher FGF23 levels.

At the systemic level, vitamins D3 and D2 are predominantly hydroxylated sequentially at position C25 in the liver and C1 in the kidney to produce biologically active 1,25(OH)_2_D_3_ and 1,25(OH)_2_D_2_. Alternative pathways exist to synthesize activated vitamin D3 or D2 via CYP11A1-derived secosteroidal hydroxylation activation in the epidermis, placenta, or adrenal gland^48–50^. These pathways are known to be modified by CYP27B1 activity according to cell- or tissuetypes. Although the physiologic role of this alternative activation in active vitamin D synthesis is unknown in the kidney transplant population, it might explain why the active vitamin D status is variable after kidney function recovery via kidney transplantation. How this alternative activation via CYP11A1 affects allograft survival or is affected by FGF23 is required to be defined in future studies.

The present study had several limitations, the first of which was its observational design, which could not exclude the possibility of residual confounding factors affecting the graft outcomes. Second, because only 400 patients with available FGF23 data were included in this study, selection bias is possible. However, there were no significant differences in baseline characteristics between the study and exclusion groups. Third, FGF23 levels were measured only at baseline and 3-year follow-up although 25(OH)D3 level, calcium, phosphorus, and PTH levels were assessed annually. Therefore, simultaneous correlations between FGF23 and other metabolic bone parameters could not be shown. Despite these limitations, this study makes a significant contribution to the field of KT by demonstrating the impact of FGF23 on longitudinal changes in 25(OH)D_3_ over a 9-year follow-up period as well as graft failure.

In conclusion, elevated pre-transplant FGF23 levels could interfere with vitamin D metabolism, even after KT, and are a risk factor for persistently low vitamin 25(OH)D_3_ and poor graft survival.

### Supplementary Information

Supplementary Information.

## Data Availability

Data supporting the findings of this study are available from the corresponding author upon reasonable request.
