# Supplementary material for: High pretransplant FGF23 level is associated with persistent vitamin D insufficiency and poor graft survival in kidney transplant patients
Source: Sci Rep. 2023 Nov 10;13:19640. doi: 10.1038/s41598-023-46889-0 (PMC10638428; doi:10.1038/s41598-023-46889-0)
Supplement: Supplementary file 1 — Supplementary Information. [file 41598_2023_46889_MOESM1_ESM.pptx]

## Slide 1
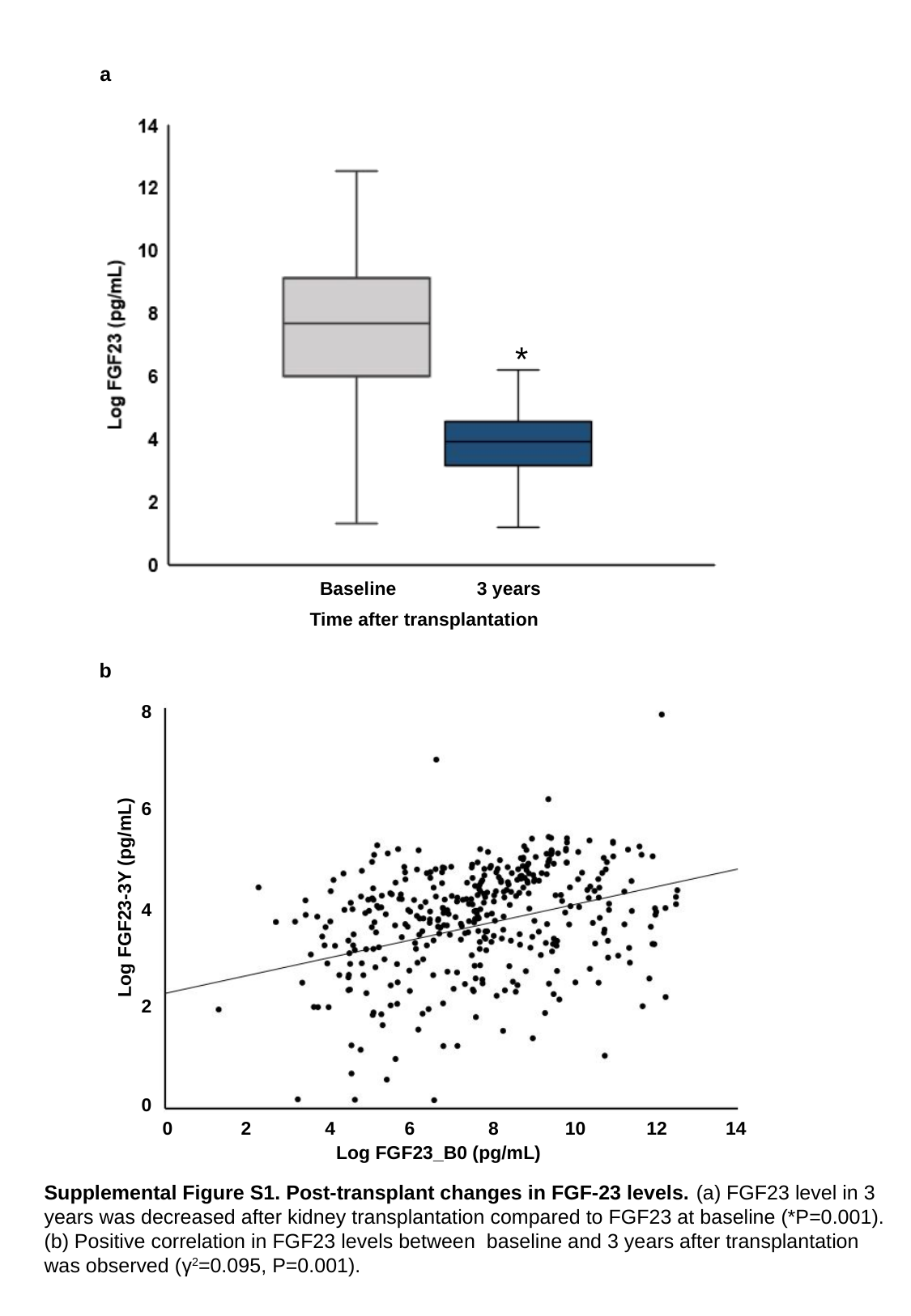

a
3 years
Baseline
*
Time after transplantation
b
8
6
Log FGF23-3Y (pg/mL)
4
2
0
0
2
4
6
8
10
12
14
Log FGF23_B0 (pg/mL)
# Supplemental Figure S1. Post-transplant changes in FGF-23 levels. (a) FGF23 level in 3 years was decreased after kidney transplantation compared to FGF23 at baseline (*P=0.001). (b) Positive correlation in FGF23 levels between baseline and 3 years after transplantation was observed (γ2=0.095, P=0.001).

## Slide 2
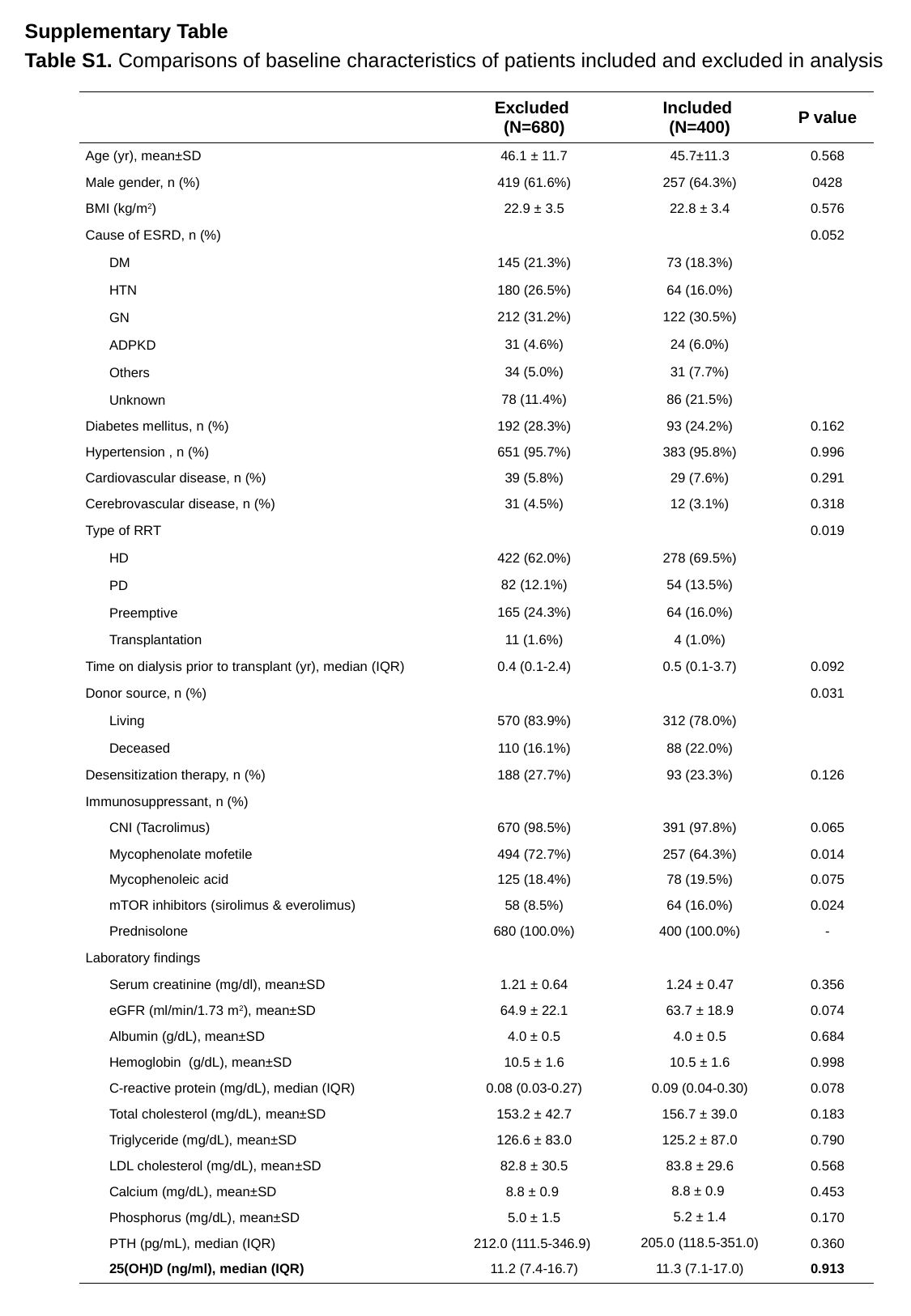

Supplementary Table
Table S1. Comparisons of baseline characteristics of patients included and excluded in analysis
| | Excluded (N=680) | Included (N=400) | P value |
| --- | --- | --- | --- |
| Age (yr), mean±SD | 46.1 ± 11.7 | 45.7±11.3 | 0.568 |
| Male gender, n (%) | 419 (61.6%) | 257 (64.3%) | 0428 |
| BMI (kg/m2) | 22.9 ± 3.5 | 22.8 ± 3.4 | 0.576 |
| Cause of ESRD, n (%) | | | 0.052 |
| DM | 145 (21.3%) | 73 (18.3%) | |
| HTN | 180 (26.5%) | 64 (16.0%) | |
| GN | 212 (31.2%) | 122 (30.5%) | |
| ADPKD | 31 (4.6%) | 24 (6.0%) | |
| Others | 34 (5.0%) | 31 (7.7%) | |
| Unknown | 78 (11.4%) | 86 (21.5%) | |
| Diabetes mellitus, n (%) | 192 (28.3%) | 93 (24.2%) | 0.162 |
| Hypertension , n (%) | 651 (95.7%) | 383 (95.8%) | 0.996 |
| Cardiovascular disease, n (%) | 39 (5.8%) | 29 (7.6%) | 0.291 |
| Cerebrovascular disease, n (%) | 31 (4.5%) | 12 (3.1%) | 0.318 |
| Type of RRT | | | 0.019 |
| HD | 422 (62.0%) | 278 (69.5%) | |
| PD | 82 (12.1%) | 54 (13.5%) | |
| Preemptive | 165 (24.3%) | 64 (16.0%) | |
| Transplantation | 11 (1.6%) | 4 (1.0%) | |
| Time on dialysis prior to transplant (yr), median (IQR) | 0.4 (0.1-2.4) | 0.5 (0.1-3.7) | 0.092 |
| Donor source, n (%) | | | 0.031 |
| Living | 570 (83.9%) | 312 (78.0%) | |
| Deceased | 110 (16.1%) | 88 (22.0%) | |
| Desensitization therapy, n (%) | 188 (27.7%) | 93 (23.3%) | 0.126 |
| Immunosuppressant, n (%) | | | |
| CNI (Tacrolimus) | 670 (98.5%) | 391 (97.8%) | 0.065 |
| Mycophenolate mofetile | 494 (72.7%) | 257 (64.3%) | 0.014 |
| Mycophenoleic acid | 125 (18.4%) | 78 (19.5%) | 0.075 |
| mTOR inhibitors (sirolimus & everolimus) | 58 (8.5%) | 64 (16.0%) | 0.024 |
| Prednisolone | 680 (100.0%) | 400 (100.0%) | - |
| Laboratory findings | | | |
| Serum creatinine (mg/dl), mean±SD | 1.21 ± 0.64 | 1.24 ± 0.47 | 0.356 |
| eGFR (ml/min/1.73 m2), mean±SD | 64.9 ± 22.1 | 63.7 ± 18.9 | 0.074 |
| Albumin (g/dL), mean±SD | 4.0 ± 0.5 | 4.0 ± 0.5 | 0.684 |
| Hemoglobin (g/dL), mean±SD | 10.5 ± 1.6 | 10.5 ± 1.6 | 0.998 |
| C-reactive protein (mg/dL), median (IQR) | 0.08 (0.03-0.27) | 0.09 (0.04-0.30) | 0.078 |
| Total cholesterol (mg/dL), mean±SD | 153.2 ± 42.7 | 156.7 ± 39.0 | 0.183 |
| Triglyceride (mg/dL), mean±SD | 126.6 ± 83.0 | 125.2 ± 87.0 | 0.790 |
| LDL cholesterol (mg/dL), mean±SD | 82.8 ± 30.5 | 83.8 ± 29.6 | 0.568 |
| Calcium (mg/dL), mean±SD | 8.8 ± 0.9 | 8.8 ± 0.9 | 0.453 |
| Phosphorus (mg/dL), mean±SD | 5.0 ± 1.5 | 5.2 ± 1.4 | 0.170 |
| PTH (pg/mL), median (IQR) | 212.0 (111.5-346.9) | 205.0 (118.5-351.0) | 0.360 |
| 25(OH)D (ng/ml), median (IQR) | 11.2 (7.4-16.7) | 11.3 (7.1-17.0) | 0.913 |
